# Nanoscale imaging of the photoresponse in PN junctions of InGaAs infrared detector

**DOI:** 10.1038/srep21544

**Published:** 2016-02-19

**Authors:** Hui Xia, Tian-Xin Li, Heng-Jing Tang, Liang Zhu, Xue Li, Hai-Mei Gong, Wei Lu

**Affiliations:** 1 National Laboratory for Infrared Physics, Shanghai Institute of Technical Physics, Chinese Academy of Sciences, 500 YuTian Road, Shanghai 200083, People’s Republic of China; 2 Key Laboratory of Infrared Imaging Materials and Detectors, Shanghai Institute of Technical Physics, Chinese Academy of Sciences, 500 YuTian Road, Shanghai 200083, People’s Republic of China

## Abstract

Electronic layout, such as distributions of charge carriers and electric field, in PN junction is determinant for the photovoltaic devices to realize their functionality. Considerable efforts have been dedicated to the carrier profiling of this specific region with Scanning Probe Microscope, yet reliable analysis was impeded by the difficulty in resolving carriers with high mobility and the unclear surface effect, particularly on compound semiconductors. Here we realize nanometer Scanning Capacitance Microscopic study on the cross-section of InGaAs/InP photodetctors with the featured dC/dV layout of PN junction unveiled for the first time. It enables us to probe the photo-excited minority carriers in junction region and diagnose the performance deficiency of the diode devices. This work provides an illuminating insight into the PN junction for assessing its basic capability of harvesting photo-carriers as well as blocking leakage current in nanoscopic scale.

PN junction (PNJ) is the active site of most semiconductor devices, such as diodes, transistors, photodetectors and solar cells^1^,^2^,^3^. Its primary role of modulating the charge carrier transport is implemented within the depletion region that mostly spans no more than several hundred nanometers^4^. Accordingly the actual electronic layout, e.g. the band alignment and the carrier profile of the junction, should be one of the key factors in optimizing the device performance^5^,^6^,^7^, particularly when it approaches the prediction limit nowadays^8^,^9^. On the other aspect, the spatially resolved electronic information could be beneficial for diagnosing the local effect^10^,^11^,^12^,^13^,^14^, such as that of the point defects, which is beyond the capacity of traditional techniques.

In the past decade, the scanning capacitance microscopy (SCM) has been widely used in the carrier profiling of various functional structures^15^,^16^,^17^,^18^,^19^. However, confident SCM analysis on PN junction remains a challenge. Firstly, the strong built-in electric field increases the difficulty to resolve the highly mobile carriers and hinders the reliable interpretation^16^,^17^,^18^,^19^,^20^,^21^. Secondly, surface charges may exist and lead to re-distribution of carriers near the tip-engaged area^20^. The situation could be more complicated on the cross section of PNJ since the polarity and density of surface charges are expected to change remarkably across the reversely doped area. Furthermore, SCM is known of detecting the local majority carriers, but the capability of probing the minority carriers in the PNJ has not been shown, which is critically wanted for developing high performance photovoltaic devices^22^.

In this work, with an improved spatial resolution of SCM on narrow bandgap semiconductor, the distribution of differential capacitance (dC/dV) along the PNJ of In_0.53_Ga_0.47_As/InP photodetector has been delicately delineated. Based on that, significant nanoscale dC/dV response to light illumination was observed in the InGaAs depletion region of PN junction. Moreover, this dC/dV variation at PNJ is closely related to the device performance. The findings, consistent with those of photoluminescence study as well as the low frequency noise analysis, suggest that defects in the InGaAs/InP interfacial region could be responsible for the performance deficiency of PNJ and device. By fitting the experimental SCM results with 2-dimensional numerical model, the density distribution of surface charges along the cross section of PNJ is obtained. The dC/dV photoresponse in the depletion region, which reflects the efficiency of PNJ to collect photocarriers, is demonstrated to arise from the recombination between surface charges and the photo-excited minority carriers.

## Results and Discussions

### Imaging the photoresponse in PN junction

**Figure 1:**
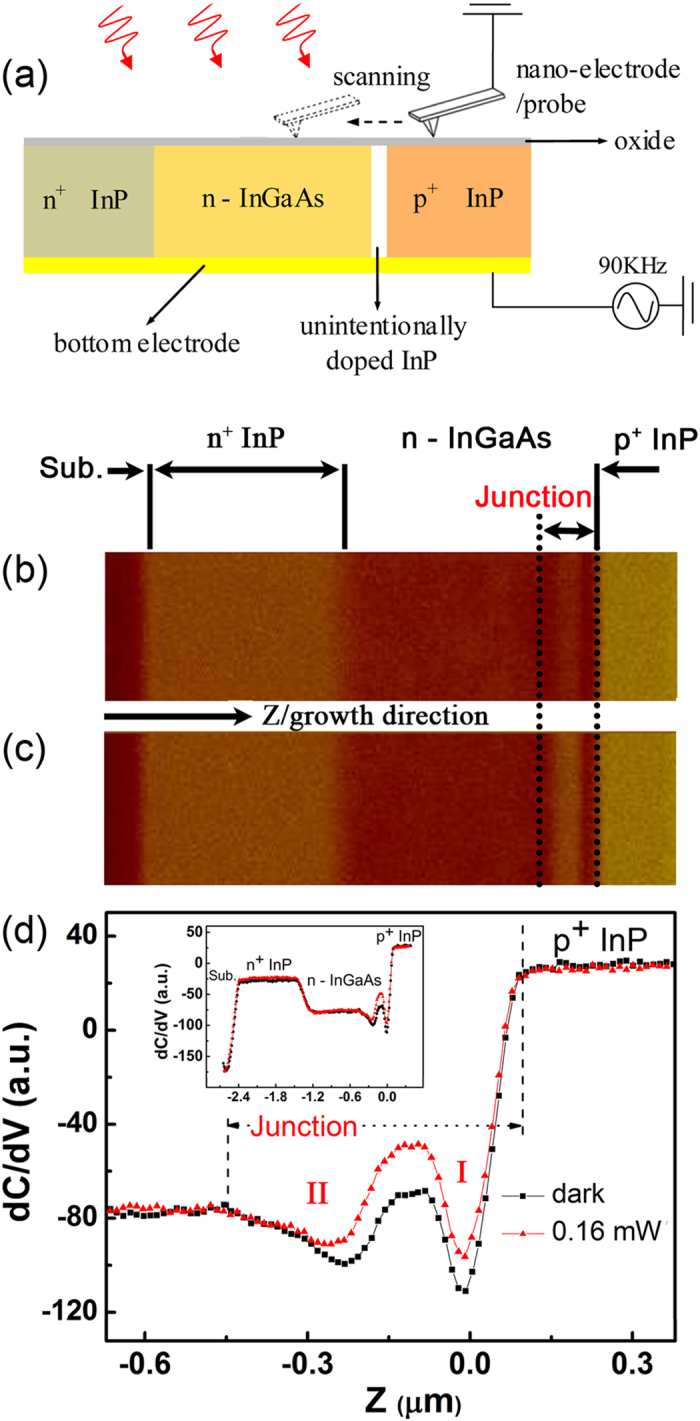
The configuration and the results of the photo-excited SCM measurements on the cross section of In_0.53_Ga_0.47_As photodetector. (**a**) The schematic experimental setup with the detail described in the “Methods” section. The SCM image of the device structure taken in dark condition (**b**) and illuminated by 808 nm laser with intensity of 1.3 mW/cm^−2^ (**c**). (**d**) The dC/dV profiles of the photodetector both in dark and under illumination. The inset shows a full view of the SCM profiles of the sample.

The examined samples are the typical InGaAs mesa photodetector, operating at a wavelength between 0.9 μm and 1.7 μm. The detector was designed to be the “n^+^ n p^+”^ structure with a 1 μm n^+^-InP (2 × 10^18^ cm^−3^) layer, a 1.5 μm n-In_0.53_Ga_0.47_As (2 × 10^16^ cm^−3^) layer, a 50 nm unintentionally doped InP layer and a 950 nm p^+^-InP (2 × 10^18^ cm^−3^) capping layer. Following this sequence these functional layers were grown on the semi-insulating InP substrate by molecular beam epitaxy. The samples were cleaved for cross sectional SCM measurement as sketched in Fig. 1a.

Unlike carriers in abrupt potential wells of low dimensional structures that can be clearly resolved^23^,^24^, PNJ provides gradual potential distribution which spans several hundred nanometers, the carriers with high mobility would respond to the tip bias from a fairly long distance. A series of improvements were then adopted to circumvent this difficulty. The conductive diamond coated Si probe was chosen for the SCM characterization due to its sharp edge at the tip end as well as the excellent mechanical and electrical endurance. The contact force between the probe and the sample was minimized to reach a small contact area. Moreover, the measurements were done in the nitrogen atmosphere to avoid the electric field induced oxidization and the humidity induced stray capacitance. With these modifications and the optimized parameters, nanoscale SCM characterization can be routinely achieved on III-V semiconductors, which offers a chance to experimentally resolve the detailed layout of junction area in narrow bandgap photovoltaic devices.

Figure 1b presents the cross-sectional SCM image of the photodetector taken in dark condition with the corresponding dC/dV distribution plotted in Fig. 1d as black line. With all the function layers clearly discriminated by their doping properties, some fine features of the dC/dV profile are disclosed in InGaAs depletion area. It can be verified that this is the characteristic appearance of PN junction since it won’t exist while the capping p-InP is etched (Supplementary Fig. 1). In principle, the local differential capacitance is expected to transit monotonically from negative to positive signal when the SCM tip scans from *n-* to *p-*type doped area. Practically, slight valley of dC/dV signal usually emerges in the depletion region due to the nonlinear sensitivity of measuring setup^17^,^18^,^20^,^21^. Nevertheless the exquisite SCM feature of PN junction presented here has not been reported including our previous studies on similar structures^25^,^26^. We attribute this progress to the improved spatial resolution and electrical sensitivity in the SCM experiments, which, together with the numerical simulation discussed later, makes it possible to investigate the performance related electronic layout in the PN junction.

SCM measurement under illumination could provide further information for the analysis of photovoltaic structures on their operating condition. Figure 1c shows the SCM image of the sample excited by laser at 808 nm. Noticeable change of SCM signal occurs in the PN junction compared with that in dark condition. Meanwhile no obvious variation could be observed for the junction width, indicating a relatively low illumination intensity (Fig. 1d)^17^. These phenomena suggest that the measurement is sensitive to the faint variation of local electronic environment induced by photo-injection.

### Numerical elucidation on the dC/dV profile

Quantitative analysis of SCM results, especially that of PNJ, suffers from the reproduction of the real electronic circumstance, including the lateral built-in electric field as well as the surface effect. Unlike the reported dC/dV profiles of Si PN junctions that could be reproduced by the numerical calculation with uniform distribution of surface states/charges^21^,^27^, here the situation is more complicated for the junction of compound semiconductors.

**Figure 2:**
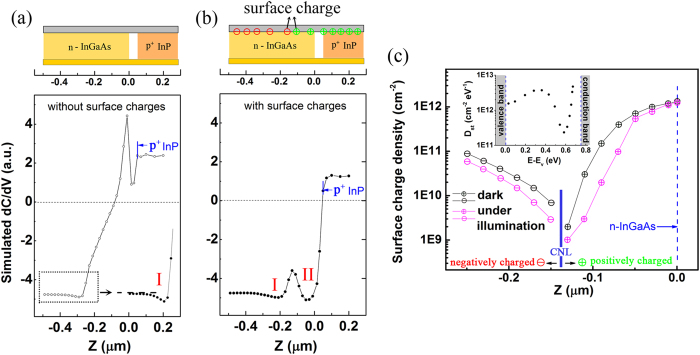
The fitting of the experimental dC/dV profile taken in dark condition. The simulated dC/dV profile of the PNJ **(a)** without surface charges, **(b)** with surface charges at the oxide/semiconductor interface. **(c)** The spatial distribution of surface charges obtained from the simulation of dC/dV profiles in dark condition and under illumination. The inset is the derived profile of surface trap density (D_st_) on In_0.53_Ga_0.47_As.

The 2D simulation started from fitting the experimental dC/dV profile taken in dark condition. The details of the numerical model could be found in the “Methods” section. As shown in Fig. 2a, when no fixed charges were set at the sectional surface, the numerical calculation could give qualitatively correct dC/dV signal on the p^+^ InP and n-InGaAs neutral region (z < −0.25 and z > 0.05 μm), but failed in predicting the feature in the junction area. Considering the fact that high density of surface states exists on oxygen adsorbed III-V semiconductors^28^,^29^,^30^, surface charges were then adopted in the simulation. Furthermore, the density of surface charges (DSC) is determined by the bulk Fermi level which will shift continuously from p- to n- region. This should result in a gradient density of fixed charges across the charge neutrality level (CNL) in the midgap.

The inference was supported by numerical simulation. As shown in Fig. 2b, the experimental dC/dV profile could be well reproduced with the spreading density of surface charges on cross section of InGaAs depletion region (Fig. 2c). The demarcation between positive and negative surface charges is found at the peak between “valley I and II” (Fig. 2b), which helps to locate the charge neutrality level (CNL) of oxygen adsorbed In_0.53_Ga_0.47_As(110) at 0.21 eV below the conduction band minimum (CBM). The density profile of surface traps (D_st_) on of In_0.53_Ga_0.47_As (110) is then derived and shown in the inset of Fig. 2c. Compared with the most studied Al_2_O_3_-In_0.53_Ga_0.47_As(100) system^28^,^29^,^31^,^32^, the oxygen-In_0.53_Ga_0.47_As(110) interface introduces relatively lower density of trap states in the bandgap, and its distribution deviates from the traditional parabolic profile when approaching the valence band maximum.

The simulation then casts light on the distinct dC/dV features in the InGaAs/InP n - p^+^ junction. For “valley I”, both the initial increase and subsequent reduction of the dC/dV amplitude, originates from the slowly decreased electron concentration along the growth direction. The reduction of dC/dV amplitude starts at the point where the majority carriers of electrons are efficiently depleted, and the contribution of holes to the differential capacitance is comparable to that of electrons, this leads the dC/dV value to approach zero. Based on these interpretations, this specific structure (valley I) is inferred as a typical SCM (dC/dV) feature of the asymmetrical p^+^- n or n^+^- p junction, which is independent of the material and the fabrication process. In contrast, the discovery of “valley II” highly depends on the experimental spatial resolution. This fine structure arises from the gradual distribution of surface charges on the cross section of the PNJ. As stated earlier, the surface of “valley II” is positively charged, which will deplete holes and accumulate electrons. The contribution of holes (or electrons) to the capacitance response decreases (or increases), thus the dC/dV signal experiences a dip when it steps into valley II, which denotes an enhanced response from electrons. The subsequent transition of dC/dV signal from negative to positive value arises from the rapid increase of the hole concentration (up to 2 × 10^18^ cm^−3^). In this region, the effect of surface charges and electrons on the capacitance response is relatively much lower.

**Figure 3:**
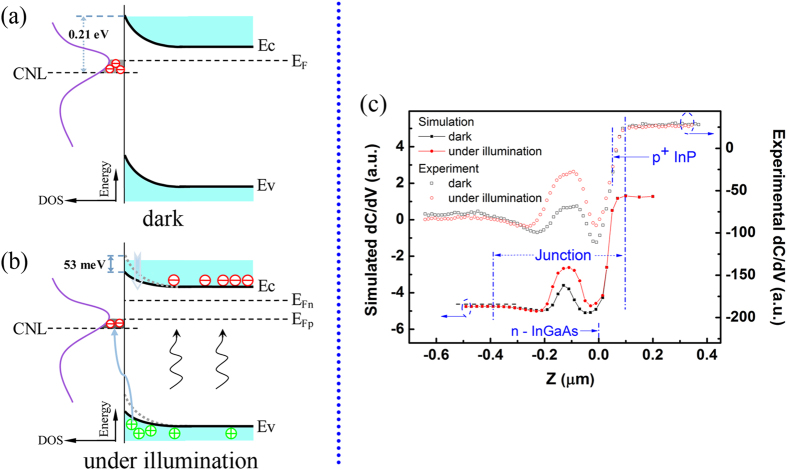
The schematic surface photoresponse process in the In_0.53_Ga_0.47_As depletion region and the fitting of the photo-excited dC/dV profile. (**a**) The schematic band structure near the surface of In_0.53_Ga_0.47_As depletion region in dark condition where the Fermi level is above the charge neutrality level. (**b**) Corresponding band structure of (**a**) under illumination of 2.8 mW/cm^2^. The surface band bending leads to the accumulation of the photo-excited holes to the surface, which will neutralize the negative surface charges. (**c**) The experimental and simulated dC/dV profiles of junction area under dark condition and light illumination.

Under illumination, the photo-excited carriers in junction area will neutralize the surface charges as illustrated in Fig. 3b. Thus with significant decrease of charge density on the sectional surface of the PNJ (Fig. 2c), the simulation shown in Fig. 3c exhibits typically the same dC/dV features as the experiment. In contrast, light illumination caused negligible change on dC/dV profile when no surface charges were set on the cross section (Supplementary Fig. 2). According to the calculation, the surface Fermi level could shift 53 meV under illumination of 2.8 mW/cm^2^, and gives rise to recordable dC/dV response in junction area.

### Performance relevance of dC/dV photoresponse at PNJ

**Figure 4:**
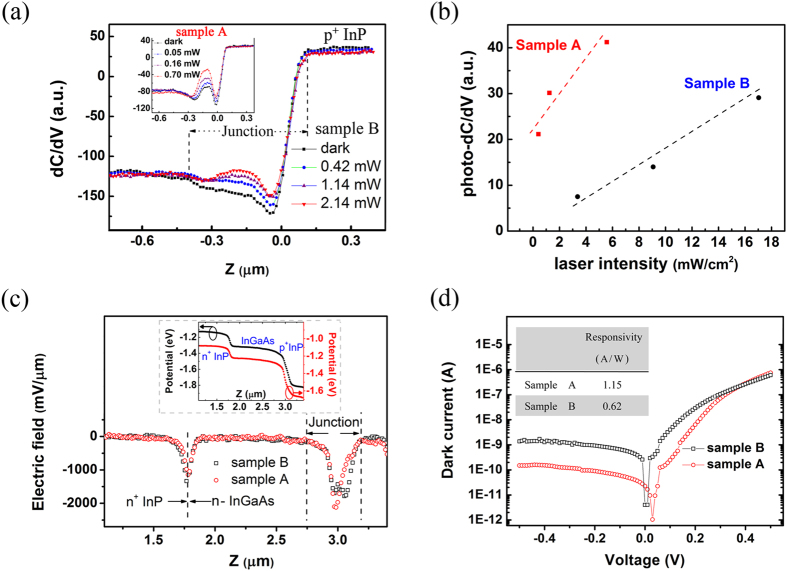
The microelectronic layout and device performance of sample A and B. **(a)** The SCM (dC/dV) profiles of sample B under illumination of different laser power, while the inset shows those of sample A. The laser spot is 4 mm in diameter. **(b)** the peak response of dC/dV signal to the excitation intensity for sample A (red scatter line) and B (black scatter line). The red and black solid curves are the guide lines to show the increasing tendency. **(c)** The electric field distributions of sample A and B, which were derived by the differentiation of the surface potential data shown in the inset. **(d)** The dark current of diode devices made of sample A and B. The inset shows the photo-responsivity of these two devices.

As the localized dC/dV response to light arises from the recombination between photo-carriers and surface charges, its magnitude can actually denote the concentration of the photo-excited minority carriers in the PNJ and further reflect on the photoelectric performance of the detector. When comparing two series of InGaAs/InP PNJs designed with same structure, one can find in Fig. 4a,b, although both junctions exhibit local response of capacitance to the illumination, the peak sensitivity of dC/dV to excitation can differ for 2.3 times in magnitude between sample A and B. This is consistent with the photoelectric property of their devices, the photo-responsivity of diode made of sample A is nearly twice than that of sample B (shown in the inset of Fig. 4d).

In general, the function of a PNJ to collect and separate the photo-carriers is determined by its build-in field. Figure 4c presents the electric field distribution of two samples along the growth direction, which is derived from the surface potential alignment (shown in the inset of Fig. 4c) measured by Scanning Kelvin Probe Microscopy. It needs to be mentioned that the contact potential difference is likely to be smeared by surface charges at the cross section; still, it could be cited as reliable evidence to evaluate the relative intensity of electrical field inside the bulk. As one can see in Fig. 4c, while similar electric field distributions were observed at the n^+^-InP/n-InGaAs interface (Z ≈ 1.75 μm) for both samples, the build-in field at PNJ of sample B shows an expanded peak and with a relatively lower peak value in contrast with that of the regular one (sample A). This, together with the dC/dV findings, could be the clue to understand the difference of photoelectric property between these two samples.

Besides the poor photoresponse performance, a weak build-in electric field could also worsen the leakage character of PNJ in dark condition. This is confirmed by the dark current property of two diodes shown in Fig. 4d. Under reverse bias, the difference of dark current reaches one order in magnitude.

**Figure 5:**
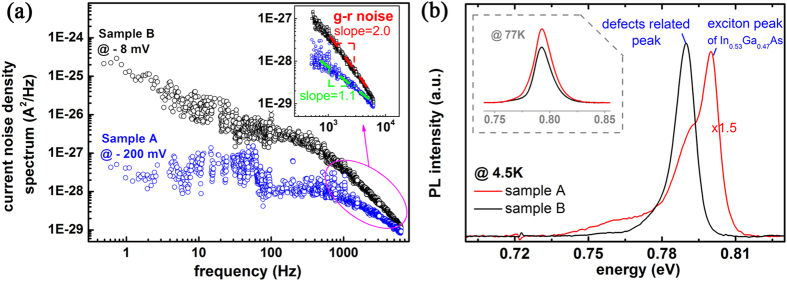
The noise and PL spectrum of sample A and B. **(a)** The noise spectrum of sample B and sample A under the reverse bias voltage of −8 mV and −200 mV respectively. The inset shows the fitting slope of spectrum in the 300–10000 Hz section. **(b)** The PL spectrum of sample A and B taken at 4.5 K. The samples are optically excited by the 647 nm laser. The PL intensity of sample A is multiplied by 1.5 to make its peak intensity close to that of sample B. Note that the integral PL intensity of sample A is larger than that of sample B. The inset gives the PL spectrum obtained at 77 K for sample A and B, both luminescent peakes are centered at 0.792 eV, indicating the same indium-gallium composition in the absorption layers of two samples.

The discovery of electronic singularity in junction area is partially confirmed by low frequency noise analysis on both diodes. As shown in Fig. 5a, the noise of sample A is substantially lower than that of sample B and show a typical 1/f style over 1000 Hz. In contrast, an obvious generation-recombination (g-r) noise is observed in sample B even at very low bias voltage, which indicates that defect-assisted generation-recombination process dominates the electrical noise in the depletion region of PNJ in sample B (more noise spectra of sample B can be found in Supplementary Fig. 3)^33^,^34^.

On the aspect of optical transition, photoluminescence (PL) study gives further clue to understand the SPM findings on junction area. Laser with wavelength at 647 nm was chosen as the incident light so that photo-carriers were mostly excited in InP capping layer; consequently recombination in the junction depletion zone would play a major role in luminescent emission of InGaAs layer. PL spectrum of InGaAs was obtained at 77 K and 4.5 K respectively (Fig. 5b). At 77 K, both samples present photoluminescence with same peak energy corresponding to the inter-band transition of In_0.53_Ga_0.47_As, which implies the identical In-Ga composition between two samples. However difference emerges at 4.5 K. While similar integral intensity of InGaAs luminescence was yielded by the two samples, that of sample A mainly comes from exciton recombination at 0.80 eV as expected for high quality InGaAs layers^35^,^36^. As regards to the PL of sample B, emission centered at 0.79 eV dominates the spectrum which is supposed to be related with the defects^35^,^36^,^37^, particularly those in the InGaAs/InP interfacial region. This could be responsible for the abnormal electrical and photoelectric properties of the PN junctions.

This study unveils the electronic layout of the In_0.53_Ga_0.47_As/InP PN junction on its cross section. It’s found that the profile of differential capacitance in the depletion region is mainly affected by the gradient distribution of surface charges. Under illumination, the recombination between surface charges and photocarriers allows us to extend the capability of SCM, which is generally referred as a tool for sensing local majority carriers, to characterize minority carriers in PNJ. By reproducing the photo-dC/dV distribution, it tells that the surface photovoltaic effect can even pull down the surface Fermi level of InGaAs depletion layer by 53 meV when excited with moderate light intensity of 2.8 mW/cm^2^.

The observed dC/dV response to light excitation in PN junction can actually denote its efficiency to collect photo-excited carriers, and shows a close relationship with the behavior of the photodiodes. Comparison between different samples suggests that defects control in the junction area is critical for photodetectors to approach its ideal performance. This study offers a way of locating and evaluating the electronic irregularity in PNJ in nanoscale. Also its high electric sensitivity and spatial resolution is well-suited for the scaling down of the photovoltaic devices.

## Methods

### The setup of the SCM and SKPM measurements

For the SCM experiments: the conductive diamond coated Si probes were used in contact mode for SCM measurements. The force constant of cantilever is 21–98 N/m. The microscopic tip radius is in the regime of 10 nm owing to sharp edges of single diamond crystals at the very end of the tip. During the experiments, the tip was kept virtual ground and served as a scanning nano-electrode, while AC bias voltage of 1.0 V at 90 KHz was applied to the bottom common electrode. An external 808 nm laser was introduced from the upper side (schematically shown in Fig. 1a) for the photo-excitation with a spot size of ~4 mm. In order to minimize the disturbance from the internal stray light, the AFM laser (670 nm) spot was set at 90 μm away from the front end of the probe’s cantilever^25^,^26^,^38^.

For the SKPM (or KPFM) experiments: the SKPM measurements were performed in the lift tapping mode. The first scan obtained the surface topography, and the second (interleave) scan extracted the potential signal on the lift height of 10 nm. Doped silicon probes with high aspect ratio were chosen for reproducible potential study. The nominal radius of tip curvature is 30 nm, and the resonance frequency of cantilever is 200–400 kHz.

### Noise PSD characterization

The noise PSD was measured by the Stanford Research System (SRS) SR760 FFT spectrum analyser. The signal (dark current) is first amplified by the DL1211 current preamplifier and then sent to the spectrum analyser. Also the biased voltage is applied to the sample by the current preamplifier. The experiments were done in a shielded room to screen the interference.

### PL experiments

The PL spectrum was obtained by the Bruker 80v FT-IR spectrometer installed with the InGaAs photodetector^39^,^40^. A helium-flow cryostat was used to held the sample at 4.5 K and 77K. The 647 nm laser was chosen for the optical excitation of the sample.

### Numerical simulation

Based on the experiment setup shown in Fig. 1a, the numerical model is established with SENTAURUS TCAD, a commercial package by Synopsys. The conductive probe is treated as a nano-electrode with the diameter of 6 nm; its scanning behavior is simulated by continuously changing the position of electrode. While the thickness and doping concentration of other functional layers are referring to their designed value, the static electric environment of the device is derived by introducing the basic drift-diffusion model, in which the carrier and electric field distribution are calculated by solving the coupled Poisson, electron and hole continuity equations. The recombination of the carriers in the devices is simulated by the Shockley–Read–Hall model^41^ with the parameters determined from literature data^42^. Furthermore, the AC modulation voltage applied to the sample is the same as that of the experiments, and the small-signal AC analysis model^41^ is applied to figure out the dC/dV response of the device. In this way, the numerical model can not only simulate the static electric environment of the junction, but also reproduce its dC/dV response under the AC modulated bias. For the fitting of the experimental dC/dV profile, the local density of surface charge is set as the only adjustable parameter.

## Additional Information

**How to cite this article**: Xia, H. *et al.* Nanoscale imaging of the photoresponse in PN junctions of InGaAs infrared detector. *Sci. Rep.* **6**, 21544; doi: 10.1038/srep21544 (2016).

## Supplementary Material

Supplementary Information
