# Supplementary material for: Nanoscale imaging of the photoresponse in PN junctions of InGaAs infrared detector
Source: Sci Rep. 2016 Feb 19;6:21544. doi: 10.1038/srep21544 (PMC4759585; doi:10.1038/srep21544)
Supplement: Supplementary Information [file srep21544-s1.doc]

Supplementary information:

**Nanoscale Imaging of the Photoresponse in PN Junctions of InGaAs Infrared Detector**

Hui Xia^1^，Tian-Xin Li^1^, Heng-Jing Tang^2^, Liang Zhu^1^, Xue Li^2^, Hai-Mei Gong^2^ & Wei Lu^1^

*^1^National Laboratory for Infrared Physics, and ^2^Key Laboratory of Infrared Imaging Materials and Detectors, Shanghai Institute of Technical Physics, Chinese Academy of Sciences, 500 YuTian Road, Shanghai 200083, People’s Republic of China.*

*Correspondence and requests for materials should be addressed to T.-X.Li (email: txli@mail.sitp.ac.cn), W.L. (email: luwei@mail.sitp.ac.cn).*

*1 Identify the characteristic SCM profile of PN junction*

As sketched in Supplementary Fig. 1, sample A was etched two times for sectional SCM measurements. After the first etching process, the residual capping layer is about 180 nm thick, and the “valley I and II” structures still exist in SCM profile. During the second etching process, the InP layer is fully removed which makes the InGaAs absorption layer exposed. Under this condition, the previous complex dC/dV features disappear. The contrast between these two SCM profiles demonstrates that the unique valleys type SCM feature originates from the PN junction. Note that the increased dC/dV signal when approaching the exposed InGaAs surface arises from the surface band bending, and the low doping concentration of InGaAs makes it more significant.

*
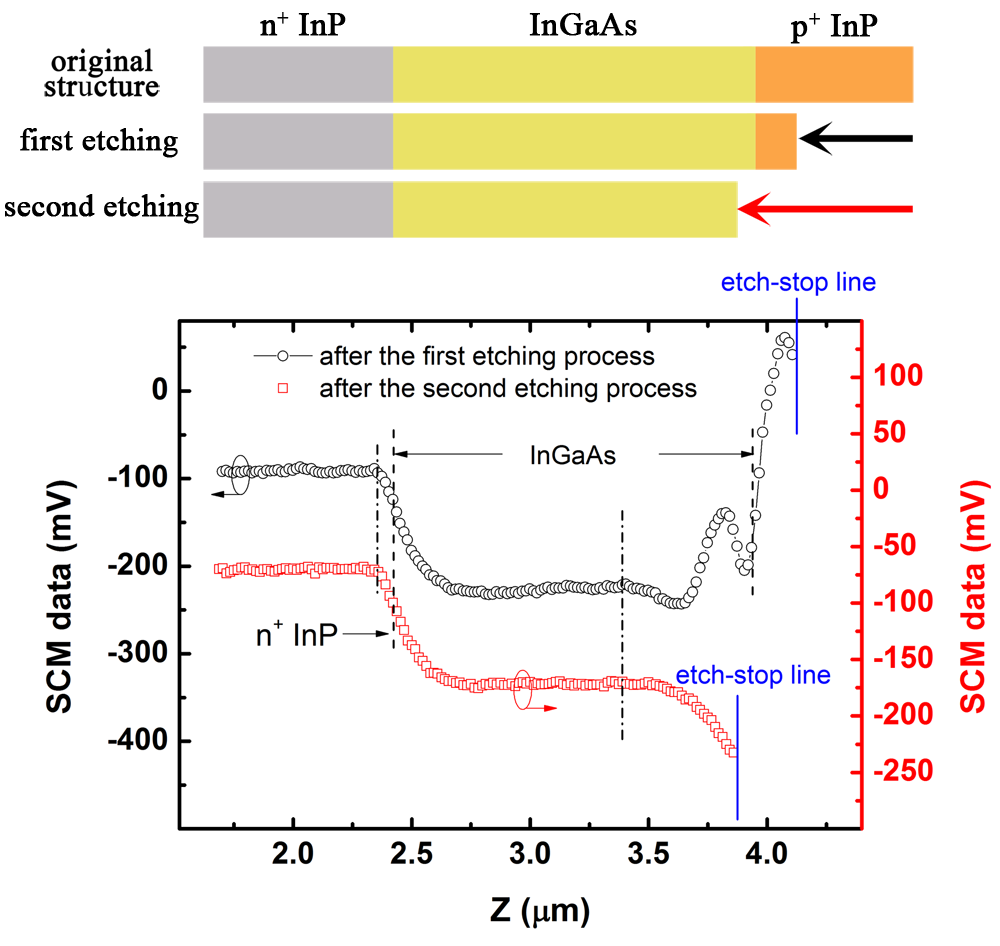
*

**Supplementary Figure 1. The schematic etching process to remove the capping layer of sample A and the subsequent SCM profiles.** The black and red scatter lines represent the SCM profiles after the first and second etching respectively.

*2 The simulated dC/dV profile without surface charges*

Supplementary Figure 2 shows the calculated dC/dV profile of the PN junction of In_0.53_Ga_0.47_As photodetector both in dark and under illumination without surface charges. Negligible change of the dC/dV signal should appear in the depletion area due to the existence of build-in electric field.


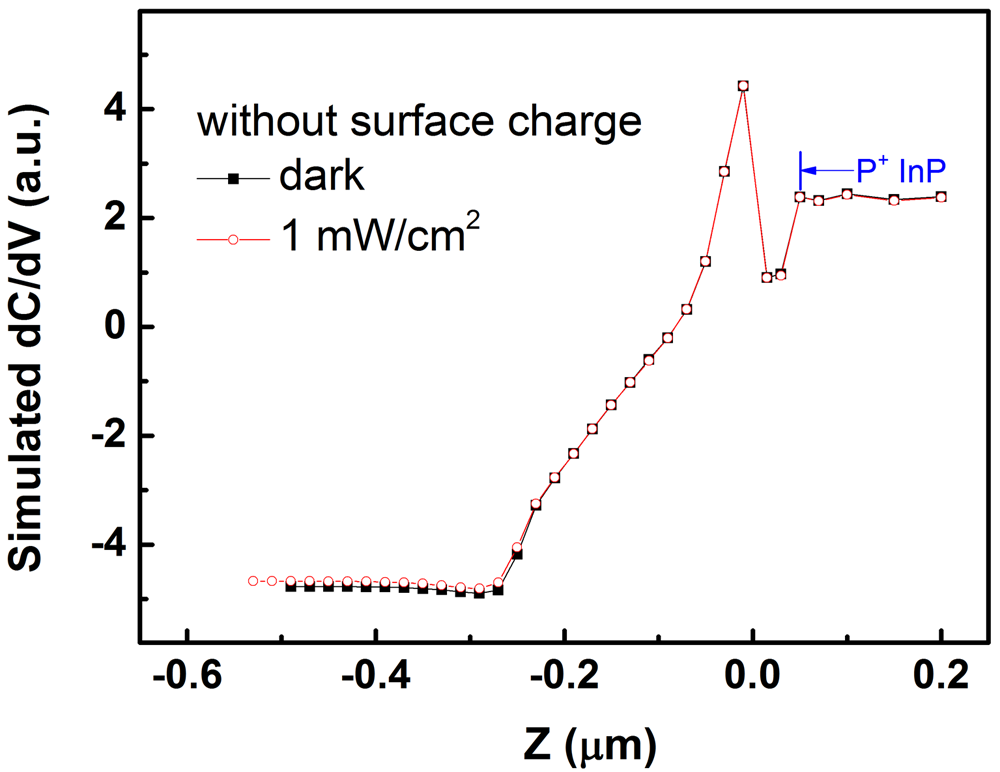


**Supplementary Figure 2. The simulated dC/dV profile of the PN junction of In_0.53_Ga_0.47_As photodetector without surface charges.** The black scatter line represents the simulated dC/dV profile in dark condition, while the red scatter line represents the simulated dC/dV profile under a illumination (@ 806nm) of 1 mW/cm^2^.

*3 The noise spectra of the photodiodes*

The noise spectrum of sample B with different biased voltages is shown in Supplementary Fig. 3. A significant generation-recombination noise can be identified even without external biased voltage applied to the device. This is in contrast with the noise spectrum of sample A (Figure 5a). Meanwhile, with the increasing of the reverse bias voltage, the 1/f noise is activated and the noise @ 0.3 Hz can reach as high as 2×10^-24^ A^2^/Hz, about 3 orders higher than that of sample A.

As shown in Supplementary Fig. 4, with comparative dark current the noise (@ 500 Hz) of sample B is one order higher than that of sample A.

*
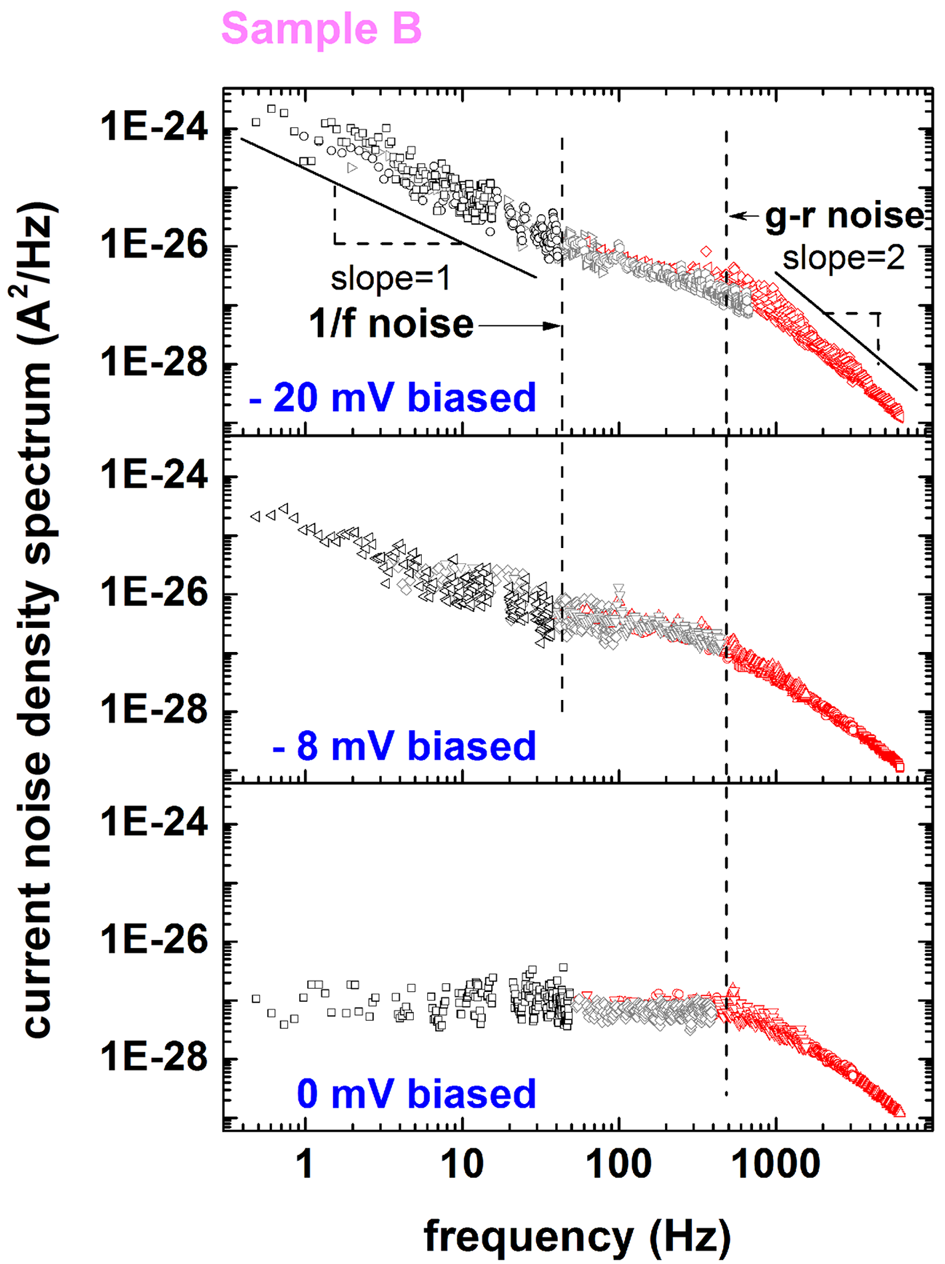
*

**Supplementary Figure 3. The noise spectrum of sample B with different bias voltages.** For f > 500 Hz, the noise spectrum shows a decreasing slope of 2.0, which is the typical feature of generation-recombination noise. For f < 20 Hz, the slope of the spectrum under both -8 mV and -20 mV biased is close to 1.0, which indicates the classical 1/f noise.


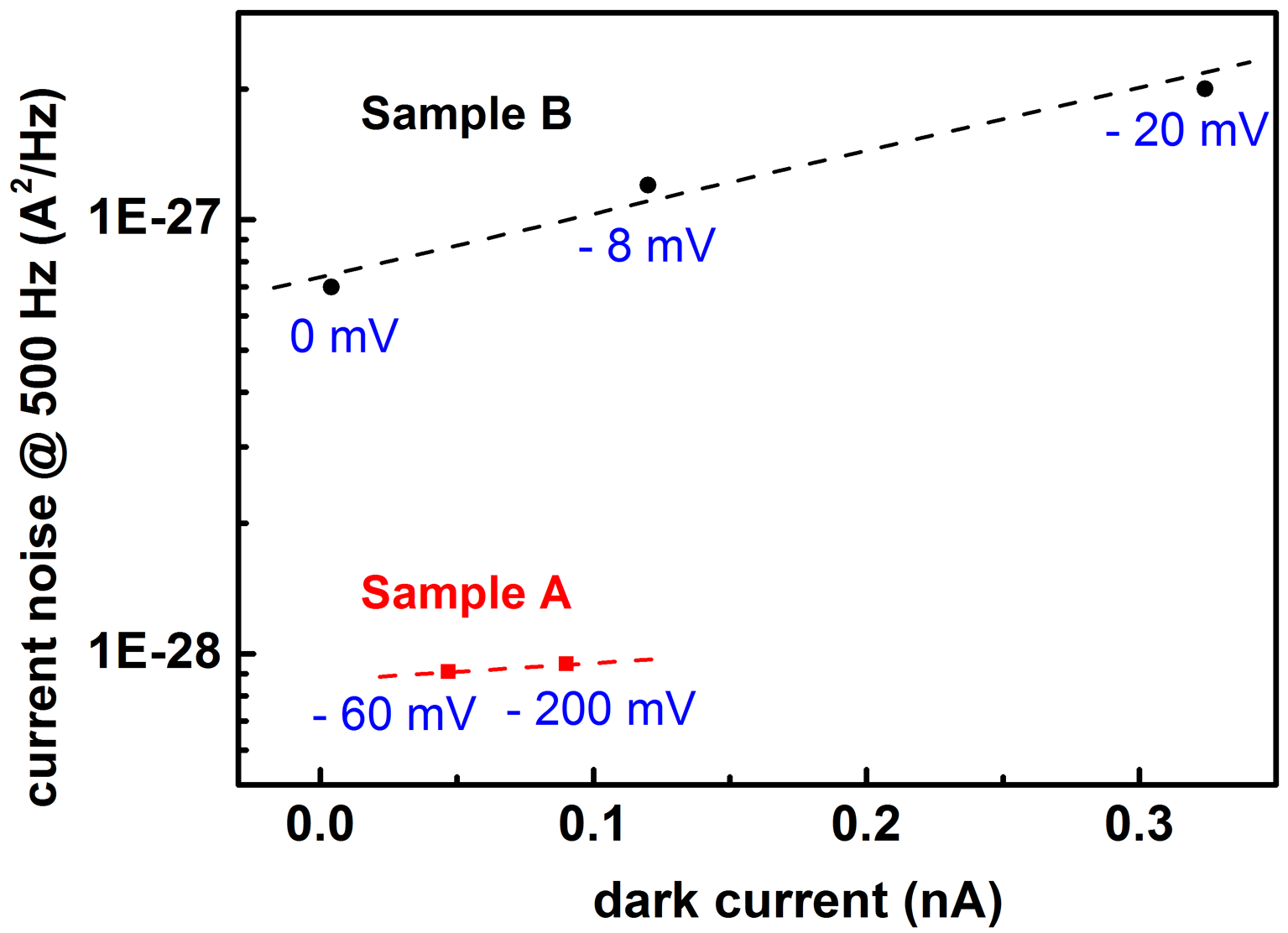


**Supplementary Figure 4. The noise (at 500 Hz) and dark current of sample A and B with different reverse bias voltage.** The black and red scatter lines represent the noise and dark current data of sample B and A respectively. The black and red dash lines are the guiding lines that shows the dependence of the noise on the dark current.

*4 The photoluminescence (PL) spectra at 4.5K*

As shown in Supplementary Fig. 5, two major features can be identified in the PL spectrums of sample A. The luminescence peaked at 0.8 eV shifts very little against the excitation density while its relative intensity increases rapidly with the laser power. Both characters are consistent with those of the exciton recombination in In_0.53_Ga_0.47_As.^3^ The lower energy photoluminescence is generally attributed to donor-acceptor recombination that is frequently observed in doped and undoped In_0.53_Ga_0.47_As/InP epilayers.^1-3^ For sample B, strong luminescence related to the shallow defect levels were observed when the excitonic emission extincts within the power range of excitation.


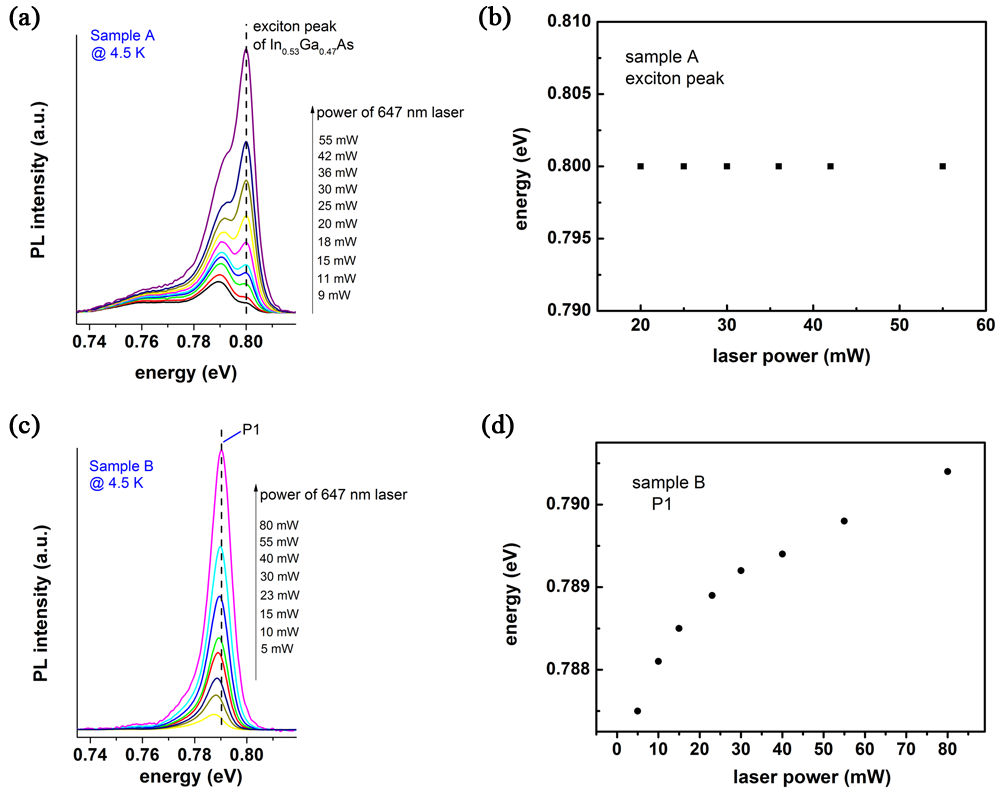


**Supplementary Figure 5. Photoluminescence spectrums of both sample A and B taken at 4.5K.** (a) the PL of sample A with different excitation powers of 647 nm laser. (b) the peak energy of exciton photoluminescence vs the excitation power. (c) the PL of sample B with different excitation powers of 647 nm laser. (d) the peak energy of feature P1 vs the excitation power.

*5 The carrier concentration of In_0.53_Ga_0.47_As absorption layer for both samples*

The electrochemical capacitance voltage (ECV) measurements were carried out to confirm the doping concentration of the In_0.53_Ga_0.47_As absorption layer. As shown in Supplementary Fig. 6, the carrier concentration of In_0.53_Ga_0.47_As layer is 2-3×10^16^ cm^-3^, while that of n^+^ InP layer is approximately 1×10^18^ cm^-3^ for both sample A and B.


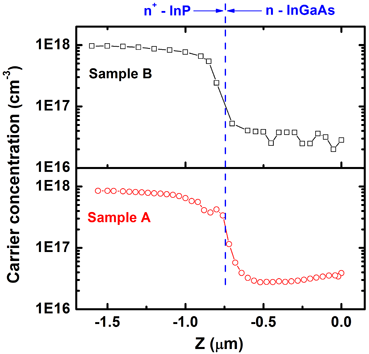


**Supplementary Figure 6.** **The carrier distributions in sample A and B.** The black scatter line represents the carrier distribution in n-InGaAs and n+ InP layers of sample B. The red scatter line represents the carrier distribution in n-InGaAs and n+ InP layers of sample A.

Supplementary References

1. Yu. P. W., Peng, C. K. & Morkoç, H. Quasi-donor-acceptor pair photoluminescence emission in Ga_x_In_1−x_As/InP. *J. Appl. Phys.* **65**, 2427 (1989).
2. Estrera, J. P., Duncan, W. M., Kao, Y. C., Liu, H. Y. & Beam, E. A. Systematic optical and x-ray study of In_x_Ga_1−x_As on InP. [*Journal of Electronic Materials*](http://link.springer.com/journal/11664) **20**, 983-987 (1991).
3. Tilly, L. P., Grimmeiss, H. G. & Hansson, P. O. Copper-related defects in In_0.53_Ga_0.47_As grown by liquid-phase epitaxy. *Phys. Rev. B* **47**, 1249 (1993).
